# An ethnozoological study of traditional medicinal animals and their products from Wolaita, Southern Ethiopia

**DOI:** 10.1016/j.heliyon.2022.e12733

**Published:** 2022-12-29

**Authors:** Abenezer Wendimu, Wondimagegnehu Tekalign

**Affiliations:** Wolaita Sodo University, Natural and Computational Sciences College, Biology Department, PO Box 138, Wolaita Sodo, Ethiopia

**Keywords:** Ethnozoology, Folk medicine, Indigenous knowledge, Medicinal animals, Traditional medicine, Zootherapy

## Abstract

**Background:**

Ethiopia has a diverse set of floral and faunal resources that are used for primary health care in traditional or indigenous medicine. However, because they are passed down orally from one generation to the next, this indigenous medical practice is being ignored and is continuing to disappear. As a result, the goal of this ethnozoological study was to assess and document traditional healers' and indigenous people's knowledge in use of animal parts or products for medical purposes in the Diguna Fango District of Wolaita, Ethiopia.

**Materials and methods:**

From March 2021 to June 2021, a field survey was conducted using personal interviews, semistructured questionnaires, and open group discussions utilizing a cross-sectional study approach. Totally, two hundred informants (125 men and 75 women) were provided information on the medicinal uses of various animals. Using a Microsoft Excel spreadsheet, the collected ethnozoological data were analyzed. The informant consensus factor, use value, and degree of fidelity were all determined.

**Results:**

More than 50 different human ailments were treated using 39 different animal parts or products. Mammals had the highest use rate (N = 26, 66.67%). In the category of treating endocrine, metabolic, and nutritional diseases, *Hystrix cristata* had a high-fidelity level (FL = 95%). The highest use value (a maximum of 1.0) was for *Bos taurus*. The disease categories with the highest informant consensus factor (ICF) values were human immune deficiency disease, reproductive health, and genito-urinary ailment categories (ICF = 1.00). Wild animals (74%) outnumbered domestic animals (26%). The most common administration route (50%) was oral, and raw remedies had the highest use reports (58.9%).

**Conclusions:**

The findings revealed that the study area contains a wealth of ethnozoological knowledge that could be useful in the formulation of novel drugs. The findings of the study should therefore be put to use in prospective ethnozoological, ethnopharmacological, and conservation-related studies in the region.

## 1 Introduction

Animals and people have depended on one another throughout history. Ethnozoology is therefore concerned with studying the past and present interactions of human civilization with animals after the first appearance of humans as a species [1]. It was reported that, of the total 252 health-promoting essential compounds, animal-based products accounted for about 8.7% [2]. Because the vast majority of the world's population relies on traditional medicinal treatments and medical practices, studying indigenous medicinal knowledge is crucial in the health-care delivery system [3].

In Sub-Saharan Africa, animal and plant-based remedies and Traditional Health Practitioners (THPs) are the primary choice of medication for the majority of people [4]. In Ethiopia alone, traditional medicines are used by approximately 80% of the population [5, 6]. In the northwestern part of the country, the medicinal uses of 51 animal species have been identified therapeutically to treat approximately 36 different ailments [7]. In the southern part of the country, 21 animal species were reported to prepare remedies for 46 different types of diseases [8]. However, the majority of ethnobiological studies carried out in Ethiopia have emphasized traditional knowledge regarding the use of plants as medicine [6, 9, 10, 11, 12, 13, 14, 15]. In the Diguna Fango District, little research has been conducted, and there is a notable absence of information regarding the use of animal products in traditional medicine.

The current study sought to describe animal use in folk medicine in the Diguna Fango District of Wolaita, Ethiopia, using an ethnozoological approach as part of a larger study project to document how animals are used by Ethiopian local people [7, 8, 16, 17, 18, 19, 20].

## 2 Methods

### 2.1 Study area description

**Figure 1:**
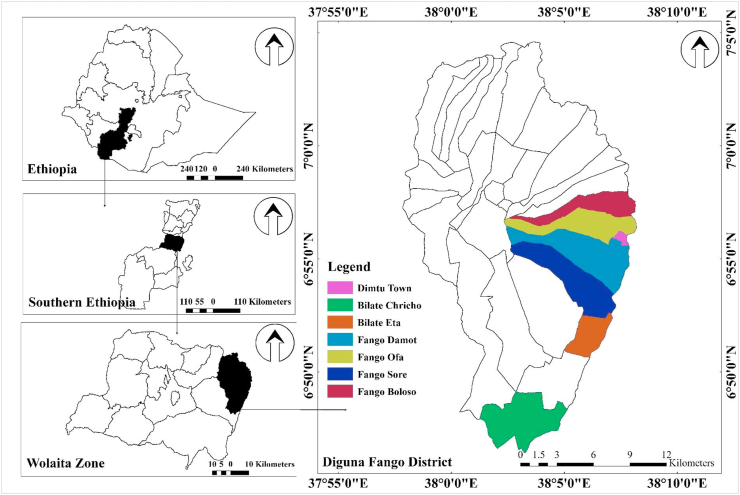
The study area map, Diguna Fango District, Wolaita Zone, Southern Nation, Nationalities, and Peoples Regional State (SNNPR) of Ethiopia.

The study was conducted in Diguna Fango District, Wolaita Zone, Southern Nation, Nationalities, and the People's Regional State of Ethiopia. The Diguna Fango District is located on the map at 6°51′37.224″–6°57′57.1″N and 38°02′15.7″–38°7′32.1456″E coordination (Fig. 1). The total area covered by the district is 371.304075 km^2^. The altitudinal range is between 1395 m a.s.l. and 2070 m a.s.l. The Diguna Fango District is located 350 km south of Addis Ababa, the capital city of the country, and 60–80 km from Sodo, the capital of Wolaita Zone. Diguna Fango is bounded to the southwest by the Damot Weyde District, to the west by the Damot Gale District, to the north by the Hadiya Zone, to the northeast by the Oromia Region, and to the east by the Sidama Region. The area's average annual rainfall and temperature are about 700 mm and 21 °C, respectively.

### 2.2 Selection of study sites

The study was carried out between March 2021 and June 2021 in seven close-by kebeles in the lower Fango cluster of the Diguna Fango District (Bilate Charicho, Bilate Eta, Fango Sore, Dimtu, Fango Boloso, Fango Damot, and Fango Ofa) in the Wolaita Zone, Southern Nation, Nationalities, and People's Regional State of Ethiopia. The Kebeles were chosen specifically for their rich and intact flora and fauna, as well as the indigenous people who used these resources in traditional medicine more frequently than in other parts of the region. However, due to practitioner fatalities, a lack of documentation, and the fact that it was primarily passed down orally, indigenous knowledge of medicinal practices is being ignored and forgotten.

### 2.3 Sampling and data collection

Thirty-five volunteers took part in field surveys to collect information about the ethnomedicinal use of animals. They were divided into five categories (seven volunteers in each group). Each group conducted the field survey in the kebeles assigned by the supervisor. The data collectors received proper training before starting the field survey. Respondents were chosen using a non-probability (volunteer) sampling technique based on their expertise in traditional medicine and experience in the preparation of traditional remedies in their society [22]. The ethnomedicinal data on the using of animals and their parts/products were gathered using the participatory rural appraisal (PRA) technique, which includes an interview, an informal meeting, and open group discussions with semistructured questionnaires, with informants also serving as investigators [23, 24].

#### 2.3.1 Discussion in groups

The importance of animals in folk medicine and related issues were discussed with the study site's selected informants after gaining informed consent before gathering data. Efforts have been made throughout the discussions to persuade the practitioners that their involvement would be advantageous to society, future generations, and the nation as a whole.

#### 2.3.2 Semi-structured interviews

Prior to the survey, an interview guide and a semistructured questionnaire were developed. The interview was prepared in Wolaitic, the indigenous language of the researcher's people, and no interpreter was required during data collection. The location and time of discussion were determined based on the preferences of the informants.

### 2.4 Data analysis

Data gathered through a semistructured questionnaire, interviews, and group discussions were cleaned, entered, and analyzed using a Microsoft Excel spreadsheet. Quantitative data were analyzed with descriptive statistics. Fidelity level, informants’ consensus factor, use values, and relative frequency of citation (RFC) were determined based on the following formula:

#### 2.4.1 Frequency of citation (FC)

The frequency of citation measures the proportion of local respondents who provided information regarding the use of particular animal species for ethnomedical purposes [25]. The FC was calculated as: FC = n/N × 100, where n is the number of times a specific species was mentioned and N is the total number of times all species were mentioned. The ethnomedicinal animals with high FC values indicate their widespread use and knowledge among the local communities.

#### 2.4.2 Fidelity level (FL)

The fidelity level is helpful in determining which species the residents prefer to heal specific types of diseases [25]. In this study, FL values ranged from 1.0% to 100%. Animals that are highly favored have a higher FL value than those that are less frequently chosen. FL values were computed as follows: FL (%) = Ip/Iu × 100, where Ip represents the total number of informants who mentioned using an animal to treat a particular ailment and Iu represents all informants who mentioned using an animal to treat any disease. It is expected that medicinal animals used frequently by most respondents for the same disease category are more likely to be helpful scientifically [25].

#### 2.4.3 Use value (Uv)

Uv signifies the use value of a specific animal used in medicine. It was calculated as: Uv = ∑Uvi/Ni, where Uvi indicates how many reports the informants had cited for that particular species, and N is the total number of respondents interviewed for a given animal species. A high use value signifies that the animal was cited by a sizable population in the research area [26].

#### 2.4.4 Informant consensus factor (ICF)

The informant consensus factor (ICF) was computed based on the reported medication for a given disease category. ICF indicates the commonly used species within a community, assisting in the selection of animals for pharmacological and phytochemical research. The reported ailments were grouped into related categories according to the works of [6, 22]. ICF values range from 0.00 to 1.00. When a single or a few animals are found to treat a specific ailment by a sizable proportion of informants, the ICF value is high. While low ICF values show that the choice of animal is a point of contention among the informants [25]. The informant consensus factor was derived using the formula ICF = Nur-nt/nur-1, where nur is the count of citations and nt is the species number used.

### 2.5 Ethics approval and consent to participate

The ethics approval was obtained from the Wolaita Sodo University Institutional Ethical Review Board (IRB) (the ethical approval number of the study is WSU 41/28/273). The methods of obtaining ethnobiological data followed the guidelines set by the International Society of Ethnobiology Code of Ethics for this research (ISE, 2006). Because signing paperwork is not usual at the study site, all respondents were given a brief explanation of the study's purpose, and oral informed consent was obtained before the interviews. The study was conducted in line with the Nagoya Protocol on Access to Genetic Resources and the Fair and Equitable Sharing of Benefits Associated with Their Use, which is an addendum to the Convention on Biological Diversity. All participants retain their right to use and author traditional knowledge, and any use of this information for purposes other than scientific publication requires further prior authorization from traditional owners as well as an agreement on access to benefits derived from subsequent use.

## 3 Results

### 3.1 Informants' demographic characteristics

During the field survey, 200 individuals (125 men and 75 women) ended up taking part in the interview and group discussion. The majority of informants (n = 94, 47%) were within the age range of 55 and above, with 73 (36.5%) being 45–54 years old and 33 (16.5%) being 35–44 years old. The majority of people in the research area lacked formal education due to the unavailability of modern education and the remoteness of educational institutions, as well as fallacious traditional personal attitudes towards modern education. Of the total respondents, 104 were illiterate, and the remaining 96 attended at least primary school, either in regular or extension programs. Most of the respondents (95%) were farmers who had a thorough understanding of ethnomedicine. Almost all study informants were not registered and organized with the modern health service delivery systems in the communities.

### 3.2 Ethnomedicinal use of animals

In the study area, 39 animal species were employed as traditional remedies to treat more than 50 different human diseases (Table 1). These animals comprised both vertebrates (32 species) and invertebrates (7 species). *Apis mellifera*, *Bos taurus*, *Capra hircus*, *Cynopterus sphinx*, *Equus asinus*, *Gallus, gallus domesticus*, *Hystrix cristata*, *Lepus fagani*, *Papio anubis*, *Phacochoerus africanus*, *Sus scrofa*, *Varanus spps*. were used to treat cancer, cold, asthma, anemia, heart disease, HIV, bacterial infections, lymphadenopathy, hypersomnia, slipped disc, bone fractures, breast pain, ear pain, fever (malaria), gastritis, malaise, paralysis, pleurisy, pneumonia, rheumatism, sciatica, skin disease, stomach pain, stunting, teeth pain, toxin, typhoid fever and others. Far more products from *Bos taurus* were reported to be used to treat 21 (40.4%) of 52 (100%) different ailments. The second place was occupied by *Hystrix cristata*, which was used to treat 15 (28.8%) different ailments.

**Table 1:** Medicinal animals, their parts, and products for traditional therapeutic purposes by the people inhabiting Diguna Fango, Wolaita, Ethiopia.

Class and local name of animals	English name	Scientific name	Parts used	Preparation	Administration route and dosage	Aliments treated	FC
**Mammals**							
Gaashuwa (W), Kerkero (A)	Warthog	*Phacochoerus africanus*	Meat	Cooked	Oral (eating the red meat with Enjera once)	Bacterial infections	44
						Rheumatism	12
			Spur, Stirrup	Warm	Topically holding the warm spur on the affected region daily until recovery	Breast pain	23
						Swelling	19
			Teeth	Warm	Topical (massaging)	Breast pain	9
			Whole body	Soup	Oral (drink soup until recovered)	Bacterial infections	21
						Common cold	63
Quxarssaa (W), Jart (A)	Crested porcupine	*Hystrix cristata*	Meat	Roasted (or preparing the meat in the form of stew)	Oral (eating the preparation with Enjera; no dosage)	Weight gain	73
						Bacterial infections	7
						Stomach pain	11
						Pleurisy	16
						Stunting	56
						Rheumatism	13
						Swelling	33
			Bone	Fumigation	Nasal inhalation (fumigating with smoke whenever feeling sick)	Malaise	17
			Whole body	Soup	Oral (drinking a coffee cup of the soup once a day)	Paralysis	5
						Stunting	28
			Bile	Raw	Oral (drinking a spoonful of bile with Enjera)	Pleurisy	13
Miizzaa (W), Lam (A)	Cattle	*Bos taurus*	Milk	Raw	Oral (drinking every day; no dose specification)	Gastritis	51
						Typhoid fever	10
						Weight gain	55
						Toxin	51
			Spleen	Raw	Oral (a day of eating three to four pieces of fresh spleen)	Pleurisy	19
						Sciatica	21
			Liver	Raw	Oral (eating the fresh liver for a day; no dose specification)	Anemia	56
			Bile	Raw	Oral (drinking half of a coffee cup or dipping in Enjera and eating)	Fever (malaria)	34
			Butter	Heated	Oral (drinking the coffee cup over a two-days interval)	Pleurisy	14
						Common cold	32
						Bone fractures	47
						Weight gain	22
			Butter	Raw	Holding on to or dressing the victim site with cold butter.	Teeth pain	17
					Heating into oil and dropping it 2–3 times a day.	Ear pain	23
			Butter	Raw	Topical application or anointing on the head; no dosage	Headache	43
			Pancreas	Raw	Oral (eating 3–5 pieces of a fresh pancreas for a day)	Sciatica	11
			Milk	Churned	Oral (drinking, no dosage)	Energy gain	44
						Teeth strength	15
			Cheese	Cooked	Oral (eating, no dosage)	Stomach pain	10
			Rumen	Raw	Oral (eating 2–3 pieces of the fresh rumen for 6–7 days)	Skin disease	18
						Swelling	8
			Digestive juices	Raw	Oral (eating with Enjera either by dipping or directly)	Fever	33
						Throat cancer	3
			Leg	Decoction	Inside a well-covered pot, the leg is cooked with spices for 4–6 days, and the soup is drunk warm)	Slipped disc	61
						Bone fractures	57
						Common cold	54
Hariya (W), Ahiya (A)	Donkey	*Equus asinus*	Milk	Raw	Oral (drinking, no dosage)	Asthma	17
						Pleurisy	26
						Pneumonia	18
			Excrement	Raw	Oral (mixing with water and drinking a spoonful of the preparation daily in the morning and evening)	Cold in chicken	2
			Excrement	Raw	Nasal (smelling; no dosage)	Sinusitis	13
						Bronchitis	9
Asaa (W), Sew (A)	Human	*Homo sapiens*	Breast milk	Raw	Topical (dropping 1–2 drops early in the morning, which is once a week)	Eye disease (children)	11
			Urine	Raw	Dropping on the victim site, no dosage	Wound	51
			Hair	Fumigation	Nasal inhalation (this method is used to determine whether or not a person is being attacked by demos. To accomplish this, use ghūl hair and smoke to fumigate a suspected for 1 min; the attacked individuals will react to the smoke and be identified; then, further action will be taken)	Evil eye (ghoul)	66
Harbbaynnuwa (W), Tinchel (A)	Ethiopian hare	*Lepus fagani*	Fur	Raw	Putting the fur topically on the wounded region, with no dosage	Wound (burnt)	69
			Meat	Roasted	Oral (eating, no dosage)	Stunting in children	44
Godariya (W), Jib (A)	Spotted hyena	*Crocuta crocuta*	Tongue	Dried	Oral (air drying, milling, mixing with water, and drinking just a cup of water once a day)	Evil eye	36
			Excrement	Raw	Oral (air drying, milling, mixing with water, and taking a spoonful once a day)	Evil eye	31
						Sciatica	11
						Stomach pain	6
			Sole	Raw	Holding hand while walking, no medication	Running problem	21
			Eye lash	Raw	No dosage when you feel like sleeping,	Over sleeping (hypersomnia)	12
			Teeth	Raw	Topical (tying around the neck)	Lymphadenopathy	5
Dorssaa (W), Beg (A)	Sheep	*Ovis aries*	Blood	Raw	Oral (drinking one glass a day)	Anemia	26
			Milk	Raw	Oral (drinking one glass a day)	Asthma	19
						Cold	39
			Meat	Roasted	Oral (eating, no dosage)	Weight gain	41
Deeshshaa (W), Fiyel (A)	Goat	*Capra hircus*	Milk	Raw	Oral (drinking one glass a day)	Fever (malaria)	17
						Tropical diseases	33
						Energy gain	48
			Blood	Raw	Oral (drinking one glass a day)	Anemia	51
			Excrement (urine and feces mixture)	Raw	Topical anointing on the victim site	Mumps	31
Wurkkaawurkkuwa (W), Yelelitwof (A)	Bat	*Cynopterus sphinx*	Blood	Raw	Topical anointing on the victim site	Skin disease	57
			Meat	Raw	Topical (massaging on the victim site)	Skin disease	55
Kanaa (W), Wusha (A)	Dog	*Canis familiaris*	Tongue	Raw	Oral (eating 2–3 pieces every day)	Rabies	32
						Gastritis	10
			Urine	Raw	Topical (either anointing or dropping on the victim site)	Eye disease	15
						Ear pain	13
						Skin disease	9
						Wart	31
Wakkallaa (W), Arjano (A)	Monitor lizard	*Varanus spp.*	Skin	Raw	Tying on the spot	Cold	3
						Rheumatism	15
Eceriya (W), Ayt (A)	Rat	*Rattus spp.*	Meat	Cooked	Oral (eating once a day, no dosage)	Stomach pain	1
Geleshshuwa (W), Zinjero (A)	Olive baboon	*Papio anubis*	Excrement	Raw	Oral (mix with water and consume one glass per day)	Fever (malaria)	12
						Eye disease	9
						Evil eye	10
			Urine	Raw	Oral (drinking a spoonful every day)	Fever (malaria)	3
Gaaraa (W), Yedur fiyel (A)	Bushbuck	*Tragelaphus scriptus*	Meat	Cooked	Oral (eating, no dosage)	Cold	17
Xadiya (W), Gumare (A)	*Hippopotamus*	*Hippopotamus amphibius*	Meat	Soup	Topical (washing the victim site with soup in a one-day interval)	Skin disease	31
Gaameelaa (W), Gimel (A)	Camel	*Camelus dromedarius*	Milk	Raw	Oral (drinking one glass a day)	Cold	48
Bo''uwa (W), Dalganbesa (A)	Serval cat	*Leptailurus serval*	Excrement	Raw	Oral (combining with water and drinking one glass per day)	Fever (malaria)	39
Guppalliya (W), Shekako (A)	Squirrel	*Xerus inauris*	Meat	Cooked	Oral (eating, no dosage)	Heart disease	26
Babbanttaa (W), Awaldgessa (A)	Aardvark	*Orycteropus afer*	Excrement	Raw	Nasal inhalation (smelling), no dosage	Evil eye	17
			Excrement	Raw	Oral (When experiencing stomach discomfort, take a full teaspoon at a time)	Stomach pain	9
			Excrement	Raw	Topical (massaging)	Skin disease	6
						Sore	6
Gudunttaa (W), Asama (A)	Pig	*Sus scrofa*	Meat	Cooked	Oral (eating, no dosage)	Fever	2
Occuwaa (W), Filfel (A)	Gopher	*Thomomys bottae*	Teeth	Raw	Tying around the gland	Swelling of gland	9
Gawaraa (W), Dimet (A)	Cat	*Felis domesticus*	Teeth	Raw	Tying around the gland	Swelling of gland	11
			Fur	Fumigation	Nasal inhalation (smelling the fur once)	Evil sprit	29
Zerussa (W), Aboshemane (A)	Leopard	*Panthera pardus*	Skin	Dried	Wearing of the skin around the victim site	Joint pain	31
					Tying on the neck	Rheumatism	9
			Tongue	Raw	Tying on the hands	Tremor	14
Parshsholiya (W),	Grey squirrel	*Sciurus carolinensis*	Meat	Decoction	Oral (milling, mixing with water, boiling, and drinking, one glass for a day)	Trypanosomiasis	41
Genessaa (W), Midakuwa (A)	Duiker	*Sylvicapra grimmia*	Skin	Dried	Tying on the legs	Walking problem in cattle	18
**Arthropods**							
Degerra (W), Tazma (A)	Sweat bee	*Halictus scabiosae*	Wild honey	Raw	Nasal inhalation	Rheumatism	38
Haattabeexiya (W), Yewuhaterb (A)	Dragonfly	*Sympetrum flaveolum*	Whole body	Raw	Tying on the neck	Swelling of gland	5
Masimasuwa (W), Gint (A)	Scorpion	*Palamnaeus swammerdami*	Whole body	Raw	Topical (milling and applying to bitten area)	Scorpions toxin	36
						Stomach pain	41
			Honey	Raw	Topical anointing	Skin disease	21
			Honey	Raw	Oral (eating a mixture of garlic, ginger, and milk; no dosage)	Erectile dysfunction (ED)	39
						Cold	59
Danqquwa (W), Mezhiger (A)	Tick	*All tick spp.*	Blood	Raw	Topical anointing on the victim site	Itchy skin disease	13
**Reptiles**							
Demiya (W), Zendo (A)	Python	*Python spp.*	Visceral fat	Raw	Fumigation or tying on hand	Rheumatism	7
						Asthma	5
						Headache	11
					Massaging on the victim site	Skin disease	2
Shooshshaa (W), Ebab (A)	Snake	*Naja naja*	Skin (shed)	Raw	Tying on the penis	Urinary problems	1
Shaaqanchchaa (W), Esist (A)	Chameleon	*Chamaeleo chamaeleon*	Tongue	Raw	Tying on the neck	Swelling of gland	12
**Birds**							
Kuttuwa (W), Doro (A)	Chicken/Hen	*Gallus gallus domesticus*	Egg	Raw	Oral (drinking 1–3 eggs every day in the morning)	Cold	55
						Sciatica	13
						Pleurisy	32
						Gastritis	44
						Common cold	13
						Abdominal pain	21
						Fever	10
			Spoiled egg	Raw	Oral (drinking one a day)	Cold in donkeys	4
			Abdominal fat	Oiled in heat	Dropping 1–2 slightly cold oil drops into the victim's ear	Ear pain	3
			Whole body	Cooked	Oral (eating, no dosage)	Weight gain	58
						Cold	11
			Excrement	Raw	Oral (mixing with water, filtering, and drinking the mixture for three days)	Stomach pain	6
			Excrement	Raw	Topical application	Skin disease	5
						Sore (head)	4
Qooraasiya (W), Kura (A)	Raven	*Corvus corax*	Egg	Raw	Oral (drinking 1–3 eggs every day)	HIV	19
						Asthma	21
Haraphphiya (W), Rigib (A)	Dove	*Columbiformes spp.*	Egg	Raw	Oral (drinking 2–3 eggs daily before meals)	Pleurisy	11
Kuraachchuwa (W), Jigra (A)	Partridge	*Alectoris rufa*	Egg	Raw	Oral (drinking 1–3 eggs in the morning every day)	Cold	46
**Fishes**							
Moliya (W), Asa (A)	Fish	*Any fish spp.*	Whole body	Soup	Oral (drinking 1 soup glass every day in the morning and evening)	Cold	63
				Oiled	Oral (drinking 1 soup glass daily)	Bone fractures	19
			Meat	Cooked	Oral (eating, no dosage)	Bone fractures	23
			Liver	Raw	Oral (eating, no dosage)	Eye disease	9

Key: FC = frequency of use citation; A = Amharic, W= Wolatic; Enjera = sour fermented flatbread made of grain, the national dish of Ethiopia and Eritrea; ghūl = an evil spirit or phantom, particularly one who is said to rob graves and feed on dead bodies. Animals were divided into their families and sorted according to their local acceptance and medication popularity in the societies.

### 3.3 Frequency of citation (FC)

The FC values for employing animal products or parts in the present study range from 1 to 73. *Hystrix cristata* (FC = 73), *Lepus fagani* (FC = 69), and *Homo sapiens* (FC = 66) were the first three most frequently cited species of animals for weight gain, wound healing, and evil eye medication, respectively. The smallest frequency of citation was observed in *Rattus* and *Python spp*. (FC = 1, for each) to cure stomach pain and urinary problems. A higher frequency of citations suggests that the majority of respondents used animals to treat a variety of ailments. A lower frequency of citations, on the other hand, indicates that the majority of respondents do not prefer a specific animal purpose to cure a variety of illnesses (Table 1).

### 3.4 Number of animal classes utilized in zootherapy practice

**Figure 2:**
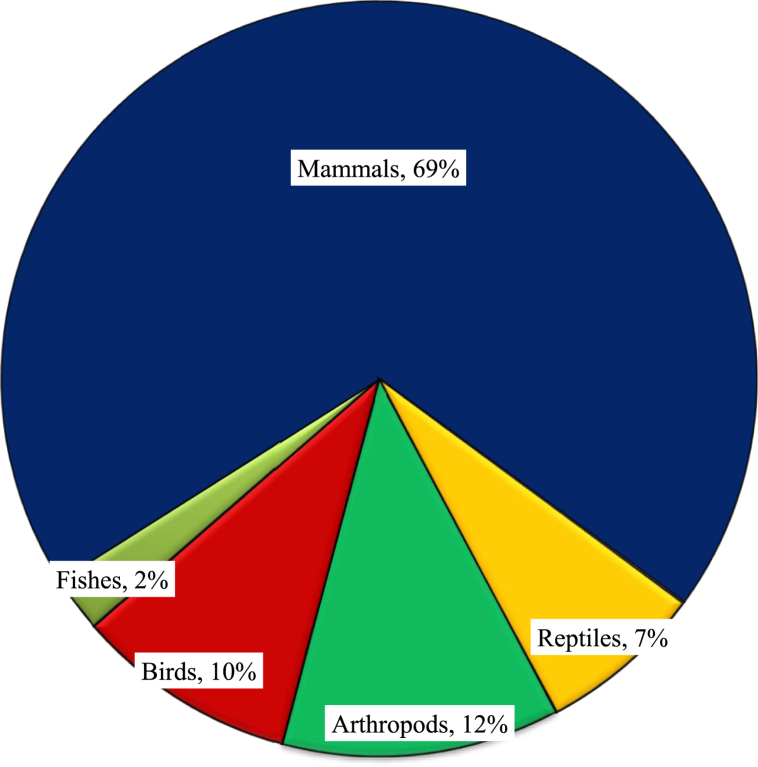
Classification of animals used in zootherapy at the study site.

Mammals were the most widely mentioned animal class in zootherapy practice (n = 26, 66.67%), followed by arthropods (n = 5, 12.81%). A further four (10.26%) avian species, three (7.7%) reptile species, and one (2.56%) fish species have all been utilized in the preparation of traditional medicines (Fig. 2).

### 3.5 Animal parts and products used in medicine

Products (honey, milk, butter, cheese, eggs) (n = 18, 21.1%), visceral organs (bile, fat, tongue, juices, liver, rumen, spleen, pancreas) 15 (17%), meat 13 (15%), and external body parts (leg, skin, sole, fur, hair, eyelash, horn, spur) (n = 12, 14%) were the top five most widely utilized remedy preparations. In addition to these, some animals’ excrement and urine, bones and teeth, exterior body parts (legs, entire body), blood, soles, fur, hair, eyelashes, horns, spurs, and larvae were used to treat a variety of illnesses in the society (Table 2).

**Table 2:** Animal parts or products used for traditional medicine in the study area.

No.	Parts or products of animals used as medicine	Citations	Percentage (%)
1.	Products (honey, milk, butter, cheese, eggs)	18	21.1%
2.	Visceral organs (bile, fat, tongue, juices, liver, rumen, spleen, pancreas)	15	17%
3.	Meat	13	15.3%
4.	External body parts (leg, skin, sole, fur, hair, eyelash, horn, spur)	12	14%
5.	Excreta (stool and urine)	10	11.7%
6.	Bone/teeth	5	5.8%
7.	Blood	4	4.7%
8.	Whole body	3	3.5%
9.	Soup	3	3.5%
10.	Larvae	1	1.7%
11.	Biting	1	1.7%

The list is based on the number of citations.

### 3.6 Method of remedy preparation

**Figure 3:**
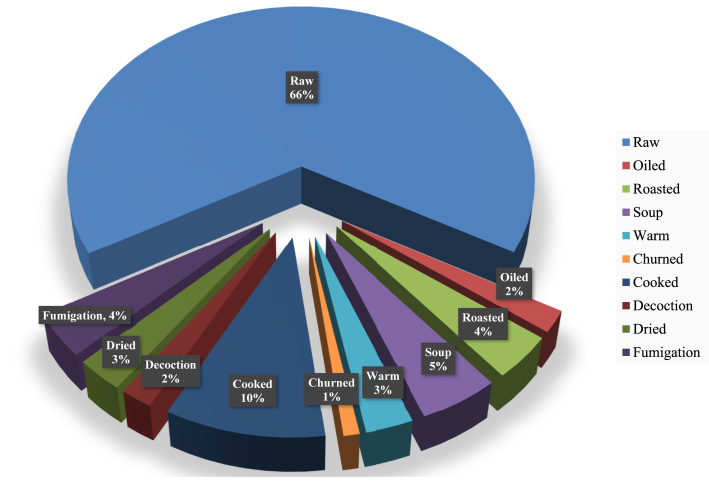
Mode of preparation of remedies.

The findings revealed ten different methods for preparing remedies. Raw remedies made up 58.9% of the overall preparation, while cooked remedies made up 9.23%, soup preparation accounted for 4.55%, and roasting and fumigation each accounted for 3.40% (Fig. 3).

### 3.7 Administration and application routes

**Figure 4:**
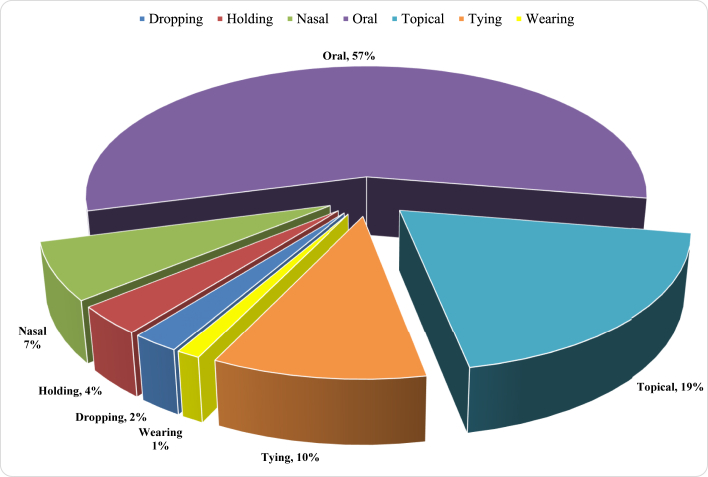
Application/administration routes.

Traditional medicine was administered in a variety of ways. The oral route, which accounted for 50% of all applications, was followed by topical (18%), tying on (9%), nasal application (6%), holding on (3%), dropping (2%), and wearing (1%) (Fig. 4).

### 3.8 Habitats of medicinal animals

In total, 39 animal species were used for therapeutic purposes at the study site, with 74% obtained from the wild and ten (26%) representing domestic animal species. This reveals that the community's traditional people and healers rely more on wild than domestic species, and it also suggests that some wild animal species in the study area were overused.

### 3.9 Informant consensus factor

Human immune deficiency disease, reproductive health, and genito-urinary ailment categories had the highest informant consensus factor (ICF) values (ICF = 1.00), followed by poisonous bites (ICF = 0.98) and cardiovascular system diseases (ICF = 0.97). When only a few or one animal species are reportedly employed by a large proportion of informants, the ICF values are higher. However, respiratory system disease (ICF = 0.77) had a lower ICF value compared to other categories. Low ICF values designate that informants use that particular animal rarely. Several animal species were utilized in treating respiratory system disorders (ICF = 32), which were then followed by general and unspecified disease categories (ICF = 19) (Table 3).

**Table 3:** Disease category, abbreviations of reported diseases, and informants’ consensus factor.

No.	Category name and abbreviations	Reported diseases	No. species	Nur	ICF
1.	Genito-urinary ailments (GUA)	Urinary problems	1	63	1.00
2.	Human immune deficiency disease (HIDD)	Human immune deficiency virus	1	19	1.00
3.	Reproductive health (RH)	Erectile dysfunction	1	50	1.00
4.	Poisonous bites (PB)	Rabies, scorpions toxin, general toxin	2	95	0.98
5.	Cardiovascular system diseases (CSD)	Heart disease, anemia	4	140	0.97
6.	Gastrointestinal ailments (GIA)	Abdominal pain, gastritis	11	154	0.93
7.	Dermatological infections/diseases (DID)	Wound, skin disease, sore	14	146	0.91
8.	Endocrine/metabolic/nutritional (EMN)	Weight gain, energy gain, stunting, walking problem in cattle, warts	12	188	0.91
9.	Sensory neuron disease (SND)	Headache, eye disease, ear pain	9	89	0.90
10.	Nervous (NS)	Hypersomnia (over sleeping), sciatica, teeth strength/pain, tremor	8	73	0.90
11.	General and unspecified (GU)	Malaise, evil spirit, evil eye, swelling, other tropical diseases, trypanosomiasis, bacterial infections,	19	169	0.89
12.	Skeletomuscular system disorders (SMSD)	Bone fracture, breast pain, joint pain, lymphadenopathy, paralysis, rheumatism, running problem, slipped disc	17	129	0.87
13.	Fever (F)	Malaria, typhoid fever	9	44	0.81
14.	Respiratory systems diseases (RSD)	Asthma, bronchitis, cold, pleurisy, pneumonia, sinusitis, throat cancer	32	141	0.77

Key: Nur = number of citations; ICF = informant consensus factor. The list is based on the informant consensus factor (ICF).

### 3.10 Fidelity level

The fidelity level calculation was made to identify species of animals that were recurrently and preferentially used by the residents of the area to treat specific ailments. The present study revealed that the fidelity level ranged from 15.9% (for *Lepus fagani* in the dermatological diseases category) to 95% (for *Hystrix cristata* in the endocrine, metabolic, and nutritional disease categories). The four animal species, *Hystrix cristata* (FL = 95%), *Bos taurus* (FL = 90%), *Gallus gallus domesticus* (81.8%), and *Apis mellifera* (FL = 80.6%), scored the highest fidelity values, whereas *Lepus fagani* scored the lowest fidelity level (FL = 15.9%) (Table 4).

**Table 4:** Disease categories and their fidelity level.

No	Animal species	Disease category	Ip	Iu	FL%
1.	*Hystrix cristata*	Endocrine/metabolic/nutritional (EMN)	19	20	95
2.	*Bos Taurus*	Skeletomuscular system disorders (SMSD)	27	30	90
3.	*Gallus gallus domesticus*	Endocrine/metabolic/nutritional (EMN)	9	11	81.8
4.	*Apis mellifera*	Respiratory systems diseases (RSD)	25	31	80.6
5.	*Homo sapiens*	General and unspecified (GU)	13	19	68
6.	*Any fish spp.*	Respiratory systems diseases (RSD)	13	22	59
7.	*Capra hircus*	Cardiovascular system diseases (CSD)	10	23	43
8.	*Phacochoerus africanus*	Respiratory systems diseases (RSD)	21	63	33.3
9.	*Cynopterus sphinx*	Dermatological infections/diseases (DID)	8	32	25
10.	*Lepus fagani*	Dermatological infections/diseases (DID)	11	69	15.9

Key: Ip = number of informants for the indication; Iu = total number of informants for animals or products; FL = fidelity level. The list is based on the fidelity level (FL %).

### 3.11 Use value

The use value (Uv) is a numerical indicator of how important a particular animal species is to the community. The highest “Uv” (maximum of 1.0) among the reported animal species was for *Bos taurus*, followed by *Gallus gallus domesticus* (Uv = 0.95), *Apis mellifera* (Uv = 0.86), and *Orycteropus afer* (Uv = 0.82). The lowest Uv of 0.12 was noted for *Papio anubis*. The high "Uv" values of these species demonstrated their extensive use in the treatment of illnesses (Table 5).

**Table 5:** Use-value of medicinal animal species for treating commonly reported diseases.

No.	Scientific name	English name	Local name	∑Uvi	Uv	% Uv
1.	*Bos taurus*	Cattle	Miizzaa (W), Lam (A)	58	58	1
2.	*Gallus gallus domesticus*	Chicken/Hen	Kuttuwa (W), Doro (A)	19	20	0.95
3.	*Apis mellifera*	Honey bee	Mattaa (W), Nib (A)	30	35	0.86
4.	*Orycteropus afer*	Aardvark	Babbanttaa (W), Awaldgessa (A)	34	41	0.82
5.	*Camelus dromedarius*	Camel	Gaameelaa (W), Gimel (A)	35	45	0.79
6.	*Hystrix cristata*	Crested porcupine	Quxarssaa (W), Jart (A)	46	58	0.79
7.	*Ovis aries*	Sheep	Dorssaa (W), Beg (A)	35	44	0.79
8.	*Any fish spp.*	Fish	Moliya (W), Asa (A)	17	22	0.78
9.	*Leptailurus serval*	Serval cat	Bo''uwa (W), Dalganbesa (A)	25	36	0.69
10.	*Felis domesticus*	Cat	Gawaraa (W), Dimet (A)	22	33	0.67
11.	*Crocuta crocuta*	Spotted hyena	Godariya (W), Jib (A)	34	53	0.65
12.	*Halictus scabiosae*	Sweat bee	Degerra (W), Tazma (A)	25	38	0.65
13.	*Python spp.*	Python	Demiya (W), Zendo (A)	18	36	0.51
14.	*Cynopterus sphinx*	Bat	Wurkkaawurkkuwa (W), Yelelitwof (A)	22	51	0.43
15.	*Equus asinus*	Donkey	Hariya (W), Ahiya (A)	16	37	0.43
16.	*Tragelaphus scriptus*	Bushbuck	Gaaraa (W), Yedur fiyel (A)	25	58	0.43
17.	*Panthera pardus*	Leopard	Zerussa (W), Aboshemane (A)	15	37	0.40
18.	*Varanus spp.*	Monitor lizard	Wakkallaa (W), Arjano (A)	20	52	0.38
19.	*Rattus spp.*	Rat	Eceriya (W), Ayt (A)	4	20	0.36
20.	*Phacochoerus africanus*	Warthog	Gaashuwa (W), Kerkero (A)	18	54	0.34
21.	*Canis familiaris*	Dog	Kanaa (W), Wusha (A)	22	69	0.32
22.	*Chamaeleo chamaeleon*	Chameleon	Shaaqanchchaa (W), Esist (A)	12	37	0.32
23.	*Sympetrum flaveolum*	Dragonfly	Haattabeexiya (W), Yewuhaterb (A)	17	55	0.31
24.	*Thomomys bottae*	Gopher	Occuwaa (W), Filfel (A)	18	64	0.28
25.	*Corvus corax*	Raven	Qooraasiya (W), Kura (A)	11	44	0.24
26.	*Sus scrofa*	Pig	Gudunttaa (W), Asama (A)	3	25	0.21
27.	*All tick spp.*	Tick	Danqquwa (W), Mezhiger (A)	9	56	0.16
28.	*Papio anubis*	Olive baboon	Geleshshuwa (W), Zinjero (A)	5	41	0.12

Key: A = Amharic language; W = Wolatic language; Uv = Use Value; % Uv = Percentage Use Value; ∑Uvi = Sum of all Use Value report. The list is based on "%Uv."

### 3.12 Threats to medicinal animals

**Figure 5:**
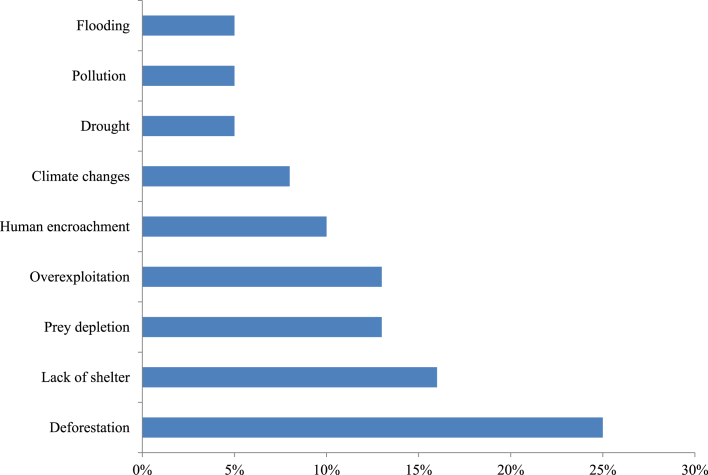
Threats to medicinal animals.

Even though people in the community used animals in traditional medical practices, anthropogenic and non-anthropogenic activities pose potential threats to animals. These activities can have a significant direct or indirect impact on the relative abundance and distribution of local fauna, including many globally threatened species. Deforestation (25%), lack of shelter (16%), prey depletion (13%), overexploitation (13%), human encroachment on wild habitats (10%), climate change (8%), drought (5%), pollution (5%), and flooding (5%), were the identified threats to medicinal animals in the study area. Deforestation is severely increasing and has accounted for the lion's share of all threats in recent days (Fig. 5).

## 4 Discussion

The World Health Organization reported that about 80% of the global population is primarily reliant on traditional medical practices that involve the use of animals [3]. Complementary and alternative medicine (CAM) has also remained a cornerstone of primary healthcare in Ethiopia, where it is still used by 80% of the population [4, 5, 6, 27]. People commonly use traditional medicines due to poverty, lack of hospitals, and because they are part of their culture [28]. The Diguna Fango District in Wolaita also uses ethnomedicine as its main form of medical care. However, no ethnomedical study was carried out in the region to document the traditionally used medicinal animals. Therefore, the aim of this study was to fill a knowledge gap in the reporting of ethnomedicine practiced in this particular area.

The majority of participants were male (62.5% of the total), with 37.5% being female. Previous studies have reported a similar pattern in other parts of Ethiopia [7, 20]. This is because, when aging, local doctors, specialists, and practitioners transfer their ethnomedicinal and magico-religious knowledge as an inheritance to their male sons or other close relatives. Therefore, a large number of male respondents available in the area were selected for interviews. The female respondents who participated in the survey were housewives who started practicing remedy preparation after careful investigation of their husbands' workings on remedy preparation materials and techniques. In other cases, some remedy preparations are generally known, and all local villagers have substantial knowledge of their usage. In fact, males hunted pest animals for food as well as medicine while keeping their farm and livestock, or purposefully for treating various therapeutic indications, which could explain our findings. Contrarily, it was reported that female respondents were more knowledgeable about using ethnomedicines than male respondents due to caring for their children and families in Khyber Pakhtunkhwa's southern area, Pakistan [21].

Participants over the age of 55 made up a sizable proportion of the sample (94%) and were primarily involved in the use of ethnomedicine. Previous studies revealed a similar pattern in Ethiopia [7, 20], demonstrating that older respondents had better expertise and understanding about ethnomedicine. There may be a variety of factors that increase knowledge with age; therefore, they should all be carefully considered. It makes sense that as people age, they have more time to learn things and, as a result, have a better understanding of medicinal animals than younger ones.

Most of the informants were illiterate (52%) and the least had an elementary level of schooling (48%), as observed in other parts of the country [20]. The significant level of illiteracy was caused by the terrible socioeconomic conditions, restricted access to learning institutions, and the fact that the majority chose traditional farming practices as a livelihood-sustaining activity.

Local communities have been known to use thirty-nine animal species for treating more than 50 different human illnesses, including cancer, colds, asthma, anemia, heart disease, HIV, bacterial infections, lymphadenopathy, hypersomnia, slipped discs, bone fractures, breast pain, ear pain, fever (malaria), gastritis, malaise, paralysis, pleurisy, pneumonia, rheumatism, sciatica, skin disease, stomach pain, stunting, teeth pain, toxin, typhoid fever, and others. Similar to this, in other parts of the country, local people used many animal species for human and livestock illnesses: in the southern region [8, 18]; the northern region [16]; and the northwestern region [7, 20]. There are similarities and cross-cultural links among ethnic tribes in the world generally and different regions of the country particularly. Therefore, in folk medicine, for the management of similar conditions, the same animal species with particular parts were recommended.

The most typically used animal species were mammals for therapeutic purposes. Similarly, studies in Ethiopia have claimed that mammals are the most widely employed species in traditional medicine [7, 8, 18, 20]. This is because mammals are plentiful sources of protein, supplemental foods, and remedies, and they were frequently utilized by the local people for meat and milk. However, in some reports, reptiles were among the animals that are most frequently utilized in ethnomedicine [1, 29].

*Bos taurus* was employed to treat anaemia, gastritis, pleurisy, sciatica, skin disease, slipped discs, and cancer. *Apis mellifera* has traditionally been used to treat erectile dysfunction, colds, and flu. *Hystrix cristata* for treating malaise, paralysis, rheumatism, and stunting; *Equus asinus* for treating asthma and pneumonia; *Lepus fagani* for treating burns; *Canis familiaris* for treating rabies; *Homo sapiens* for treating the evil eye (ghoul); and etc. were identified in the study area. Products (honey, milk, butter, cheese, and eggs), visceral organs (bile, fat, tongue, juices, liver, rumen, spleen, and pancreas), meat, and external body parts (legs, skin, soles, fur, hair, eyelashes, horns, and spurs) were widely utilized groups. Similar findings from different regions of Ethiopia reported the same things [7, 8, 10, 17]. This resemblance may be due to people's shared cultural knowledge as well as traditional medicinal animal parts and products marketed across the country.

Inhabitants in the study area were prescribed raw milk to drink if someone accidently took in toxic chemicals. They believe that the toxic chemicals will be drawn out of the body when the patient drinks raw milk. This finding is reported as novel for the first time in the current study. The leg of a *Bos taurus* (below the knee, up to the hooves) was fine cooked in a large pot with spices for three to four consecutive days, and drinking the soup directly helps to relieve colds and is a popular treatment for bone fractures and slipped discs in humans. Despite its critical therapeutic importance, this discovery is also novel and has not been reported elsewhere.

Pisces were employed to treat colds, bone fractures, and eye disease. Omega-3 fatty acids from fish contribute to the visual development and health of the retina in the back of the eye and have bone-boosting benefits [30]. Fish are also a great source of vitamin D, perfect for curing sniffles during the cold and flu seasons [30]; NPI, 2019).

The eggs of *Corvus corax* and *Gallus gallus domesticus* were employed to treat colds, sciatica, pleurisy, gastritis, abdominal pain, and fever. Previously, eggs were employed to treat similar ailments in Ethiopia [7]. It was also reported that the egg of *Gallus gallus domesticus* was effective in treating weakness, cough, and nasal congestion, as well as stopping bleeding [21], diarrhea [31], and increasing potency and libido [32]. Urinary stone treatment has found success on eggshells [21]. Similar to this, eye disorders have been treated with *Struthio camelus* egg shells [32]. According to research, eggs contain vital bioactive compounds, lipid-soluble vitamins (A, D, E, and K), omega-3 fatty acids, and minerals including folic acid, phosphorus, selenium, amino acids, and iron that contribute to their ability to have healing properties [33].

Fats and oils from animals were utilized to treat rheumatism, asthma, and headaches. Previous studies reported similar things in Ethiopia [20]. Ethnobiologists have documented that fats are used to treat neurological disorders, atherosclerosis, thrombosis, and aging effects [34, 35]. This may be due to omega-3 fatty acids in vertebrates’ fats [36]. Fats, on the other hand, are regarded as contributing significantly to heart disease, obesity, and weight gain. This is due to the high concentration of saturated fatty acids in them [33]. Therefore, using animal fat or oil could have negative effects and possibly result in health problems.

The bones of *Crocuta crocuta*, *Felis domesticus*, *Hystrix cristata*, *Thomomys bottae*, and *Phacochoerus africanus* were utilized for treating malaise, reducing gland distension, lymphadenopathy, and breast pain. However, in other studies, bones from *Alectoris chukar*, *Columba rupestris*, *Coturnix japonica*, *Otus sunia*, and *Trochalopteron lineatum* have been utilized to cure and control blood chemistry, heart problems, memory loss, renal difficulties, stomach problems, weakness, whooping cough, and wounds [18, 20]. They also lessen bone fractures (Hall, 2011). The healing properties of bone may originate from collagen fibers, inorganic minerals like calcium and phosphate, and up to "95%" of the elastic protein contained therein.

Honey was utilized for treating skin disease, erectile dysfunction, common cold, and rheumatism. Previously, it was reported to treat a variety of ailments in nanomedicine and acts as an anti-apoptosis and antiproliferative agent (Oroli, 2009 [37–39]; an anti-diabetic; an antioxidant [40, 41]; an antibiotic; an anti-cataract; an anti-inflammatory; an antifungal; and an endophthalmitis [42, 43, 44]. Studies have shown that honey is composed of sugars [45], water, proteins [46, 47], amino acids [48], vitamins [49], minerals [50], organic acids, phenolic compounds [51], and solid particles [52], as well as volatile compounds help out to have a healing property [53].

Scorpion appears to be used far more frequently in folk medicine. The entire body of the scorpion *Palamnaeus swammerdami* was milled and applied to the victim areas to treat scorpion venom-affected skin. In Korea, scorpions are used to treat a variety of medical conditions, including convulsions, facial paralysis, speech disorders, liver disorders, high blood pressure, and pain throughout the body [54]. The main active components of scorpion medicine are tri-methylamine, butotoxins, lecithin, taurine, betain, and cholesterine [54]. The venom has been analyzed and found to contain numerous valuable bioactive compounds that can explain the Chinese use of scorpion venom in connection with meningitis, epilepsy, stroke, and rheumatic diseases [55].

Raw preparations accounted for the vast majority of medications used. The same trend was observed among different tribal communities [1, 7, 8, 24, 56]. The preparation and eating of uncooked raw meat have been heavily influenced by cultural and religious factors [57]. Raw meat consumption, however, may increase the risk of humans contracting various parasites and diseases [24]. Zoonotic illnesses may spread through close contact with animals as well as through the consumption of animal products in food and medicine. Raw milk consumption, for example, has been associated with parasitic toxoplasmosis infection previously and is now prohibited. Salmonella infection can occur in a variety of organs and tissues, including bone and bile, resulting in persistent diarrhea and endotoxic shock [58]. When handling and using animal tissues from unidentified origins as medicines, the risk of transmission of additional severe and pervasive zoonoses like rabies or tuberculosis should be taken into account.

Most liquids and solids were given orally, but some were applied topically, as seen in other studies [7, 10, 59]. Topical application of medications to treat skeletal and muscular system illnesses such as rheumatism, paralysis, swelling, and arthritis was consistent with other similar findings (Jaroli et al., 2010; [7]. To cure bone fractures, joint pain, muscle aches, piles, and wounds, ethnomedicine dermal treatment is still incredibly effective (Jaroli et al., 2010; [24].

Honey, oil, milk, sugar, salt, colour, and water were utilized as additives in ethnomedicine concoctions, as in an earlier study by [7]. Although there were instances where such additives were employed, the majority of treatments did not call for the inclusion of ingredients like sugar, water, butter, honey, teff and millet flour, salt, spices, milk, eggs, or coffee. These components improve solubility, soften the taste, and make it simpler to take ethnomedicine.

During the fumigation procedures, medicinal fumes were let into the body by the nose, and various animal parts, including bones, skins, and teeth, which were believed to have healing characteristics, were tied to the neck, hands, legs, and other parts of the body.

The length of medication and dosage of remedies in the study area were not standardized. The amount of remedies for all types of health problems is determined by the practitioner who prepares the medicine and the frequency with which medications are taken up until the final recovery day. In different doses for treating similar conditions, the same animal species with particular parts were recommended. One of the major disadvantages of traditional medicine, according to various sources, is the lack of standardization and quality regulation. Furthermore, the dosage varies from one part or product to the next, and some traditional healers give different dosages and frequencies of application based on age, gender, and other conditions or vary the medicine itself based on such differences. Sometimes, even among the informants, different dosages are recommended for treating the same medical issues. Depending on the patient's age, the estimation was used to prescribe liquid remedies in the form of a full, half, or one-fourth of a coffee cup, a glass of water, a tea or soup spoon, etc. For certain remedies that are considered harmless, the doses depend on the patient's interest and/or ability to drink or eat for a certain health condition and are described as “no dosage” in the document.

### 4.1 Threats to medicinal animals

Human-animal interdependence has both positive and negative consequences. On the positive side, there are a number of societies that have a deep respect for animals. Anthropogenic and non-anthropogenic activities, on the other hand, have direct and indirect effects on the local fauna as well as increased risks to wildlife, including several globally threatened species. Deforestation (which is severe and accounts for the lion's share of all threats), overexploitation, climate change, drought, and pollution were the identified factors.

The Ethiopian Wildlife Conservation Authority (EWCA) was founded as part of the Ministry of Culture and Tourism, and new policies and legislation were drafted and implemented to successfully safeguard biodiversity, ecosystems, and ecological processes from negative human impact. The authority strictly prohibits wildlife hunting and killing unless and until authorized by legal bodies in accordance with the country's policies and legislation. On the other hand, these rules usually fail due to a lack of training provided to locals on how to protect animals and use protected areas and national parks in a sustainable manner. To protect wild animals, community service providers and governing bodies should create community awareness campaigns emphasizing alternative treatment options, such as the use of medicinal plants instead of animal products.

## 5 Conclusion

The findings reveal that the native people of Wolaita, Ethiopia, have considerable knowledge about the preparation of ethnomedicines from faunal resources. This is the first attempt to document animal-based medications from the region and provides an ethnobiological database for pharmacological, phytochemical, and synergistic studies. The locals have close ties to the numerous animal species in their habitat, and they use them in their primary healthcare system to cure a variety of illnesses. Scholarly investigation into the medical role of animals and their products should not be neglected as it is eroding rapidly due to the deaths of elder traditional healers and specialists. Furthermore, animal products and parts with the highest FL and ICF should be further studied in the future to assess the pharmacologically active ingredients by using in vitro and in vivo assays to aid in improving local people's health and spreading awareness about the management and sustainable use of indigenous knowledge.

## 6 Recommendations

Traditional medicine is used by a large proportion of both urban and rural residents in the study area. This suggests that traditional medicine plays an important role in bridging the gap between modern health services and assisting in the replacement of costly treatments and pharmaceuticals that are not readily available to consumers in the area. As a result, we made the following suggestions:

✔ Other interested researchers will need to do increasingly in-depth research on the analysis of active components and other pharmacological and ethnozoological characteristics of animals in Wolaita by using this baseline data as future references.
✔ A comparative study of ethnomedicinal animal population abundance in different geographic areas should be done to determine the local effect of traditional medicinal techniques on wildlife.
✔ To safeguard wild animals, local advocacy groups could organize community awareness campaigns that highlight alternative complementary treatment approaches, such as the use of medicinal plants rather than animal products.

### 6.1 Limitations

Ideally, field research would take more than a year to capture the whole faunal and cultural cycle of the studied area. There is also seasonal variation in both illnesses and the availability of treatments. However, one noted weakness of the current study is that it only covers a fraction of a year to obtain the necessary data.

## Consent for publication

This manuscript doesn't contain any person's data, and further consent for publication is not required.

## Availability of data and materials

The datasets generated and analyzed during the current study are included in the body of this paper.

## Funding

The study received no funding from government, commercial, or non-profit financing organizations.

## Authors’ contributions

Abenezer Wendimu: Conceived and designed the experiments; Performed the experiments; Analysed and interpreted; Contributed materials, analysis tools or data; Wrote the paper.

Wondimagegnehu Tekalign: Conceived and designed the experiments; Performed the experiments; Analysed and interpreted the data; Wrote the paper.

## Uncted Reference

[38]

## Competing interests

There is no conflict of interest between the authors regarding this paper.

## List of abbreviations

✔ NPI: National Fisheries Institute
✔ ISE: International Society of Ethnobiology
✔ PRA: Participatory Rural Appraisal
✔ THP: Traditional Health Practitioners
✔ WHO: World Health Organization
